# Push notifications for critical labs results: a pilot study in the intensive care unit (ICU)

**DOI:** 10.1093/jamiaopen/ooad058

**Published:** 2023-08-16

**Authors:** Bhavin B Adhyaru, Glenn Hilburn, Mindy Oberg, Karen Mann, Daniel Wu

**Affiliations:** Department of Medicine, Emory University School of Medicine, Grady Health System, Atlanta, Georgia, USA; Grady Health System, Atlanta, Georgia, USA; Grady Health System, Atlanta, Georgia, USA; Department of Pathology & Laboratory Medicine, Emory University School of Medicine, Grady Health System, Atlanta, Georgia, USA; Department of Emergency Medicine, Emory University School of Medicine, Grady Health System, Atlanta, Georgia, USA

**Keywords:** electronic acknowledgment, ICU, push notification, EHR

## Abstract

**Objective:**

We developed a push notification allowing for an electronic acknowledgment of critical lab results to providers in the intensive care unit.

**Materials and Methods:**

This project was conducted over a 3-month period at a large academic safety net hospital. A push notification and acknowledgment system were created to comply with the existing critical results notification requirements. We monitored the number of acknowledged results, time to acknowledgment, and lab type.

**Results:**

Prior to the push notification, lab services paged the provider. This resulted in many critical lab results relayed to the clinician beyond the expected 10-minute window. With the push notification workflow, we found that, during the 3-month period, 82, or 5.8%, of the 1414 results were acknowledged. This represented 82 less pages/calls lab services had to make.

**Discussion:**

The push notification alert was easy to use and there was quicker results notification when acknowledged. There were limitations due to hand-offs for clinicians and some were not familiar with the mobile technology and the electronic acknowledgment.

**Conclusions:**

Although the acknowledgment rate was low, every electronic acknowledgment saved lab service technicians an average of 10 minutes compared to the existing workflow. As familiarity with the technology and workflow increases, this novel form of communication has the potential to have significant cost savings for lab services, in addition to efficiency gains for lab, clinicians, and more timely care. The integration of health information technology and push notification of critical labs should be the focus of investigation for further future research.

## INTRODUCTION

Adoption of the electronic health record (EHR) has increased tremendously in the past few years.^1^^,^^2^ In 2021, 96% of nonfederal acute hospitals and 78% of office-based clinics have adopted an EHR.^3^ The EHR has many advantages including increased efficiency, improved clinical workflow and communications, and improved quality and patient safety.^1^

As healthcare becomes more reliant on EHRs, there is unique opportunity to deliver patient care with the use of clinical notifications or “push notifications” with the counterbalance of alarm fatigue. Prior literature on use of notifications of critical laboratory findings is not current, often used asynchronous systems and may or may not have directly engaged the primary clinician. Additionally, the notification did not necessarily integrate with the EHR.^4–6^ There is 1 recent study that evaluated push alert notifications of troponin results to physician smartphones for emergency room patients with chest pain.^10^ The authors found that the push alert led to quicker discharge without impacting the ED length of stay. A recent literature review in the area showed that while evidence exists on laboratory notification systems, there are very few studies since the advent of EHRs.^1^

There is potential for patient harm when clinicians fail to respond to laboratory results in a timely manner.^7^ Time lag between laboratory results availability and action is well documented.^6^^,^^8^ Traditionally, critical lab values are communicated via phone call (or page) from laboratory technicians to health care providers; however, this approach has limitations.^4^^,^^8^^,^^9^

The objective of this study is to use electronically generated push notifications to alert intensive care unit (ICU) patient’s providers of critical lab results within the EHR. In our study, we define a critical lab or result as an abnormal value that poses immediate harm to a patient and requires immediate action by the ordering provider. The labs and corresponding abnormal values are determined by our clinical laboratory leadership. Examples include blood glucose (<50 or >500 mg/dL), potassium (<2.5 or >6 mEq/L), lactic acid (>2 mmol/L), and hemoglobin (<6 or >20g/dL) abnormalities. The setting for this project is an ICU unit of a large academic safety net hospital, the Grady Health System (GHS). The target audience for the push notifications was clinicians, mainly resident physicians across 2 schools of medicine, Emory University School of Medicine, and Morehouse School of Medicine.

## MATERIALS AND METHODS

This pilot project timeline was prior to the COVID pandemic in 2019 at GHS, which is a 900+ bed level 1 trauma center. The hospital sees over 700,000 patients a year and patients come from all parts of the state. The hospital is the fifth largest public hospital in the United States and is located in Atlanta, Georgia. This hospital has a special commitment to providing primary care to underserved patients affected by barriers related to social determinants of health.

This project was conducted over 3 months (April-June 2019) in the medical intensive care units which is comprised of 40 beds over 2 ICU units. We chose this setting due to high volumes of critical laboratory results. The ICU teams include teaching teams comprised of an attending, a senior resident, and 2 interns. The ICU teaching teams are inclusive of Emory and Morehouse Schools of Medicine providers. GHS’ hospital uses EPIC’s EHR, and its mobile app, Haiku, for the intervention. For this project, GHS was using the EPIC November 2018 version.

We evaluated existing workflows; following, we modified the existing workflow to reduce variation and standardize the process. We also incorporated the push notification of critical lab results. We developed educational materials that were distributed to the ICU teams and lab service technicians. Then, we monitored the workflow and processes over the pilot period, specifically noting the number of critical results that were both sent and acknowledged, as well as the types of lab results (eg, lactic acid, troponin, complete blood count) that were included in the push notification.

## INTERVENTION AND RESULTS

**Figure 1.**
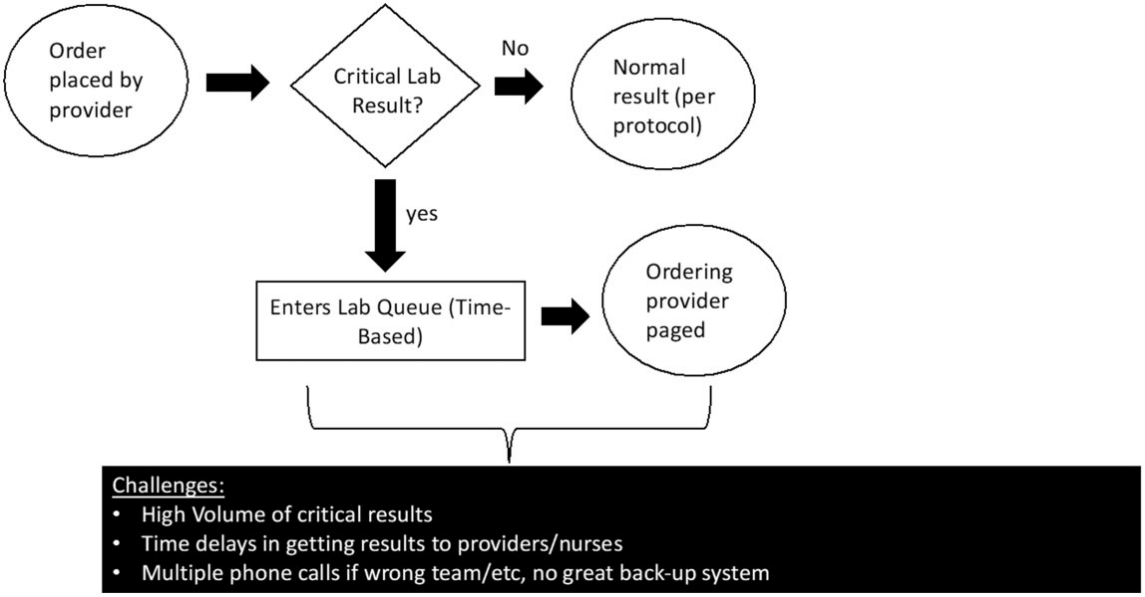
Workflow in current state prior to implementation of push notifications.

**Figure 2.**
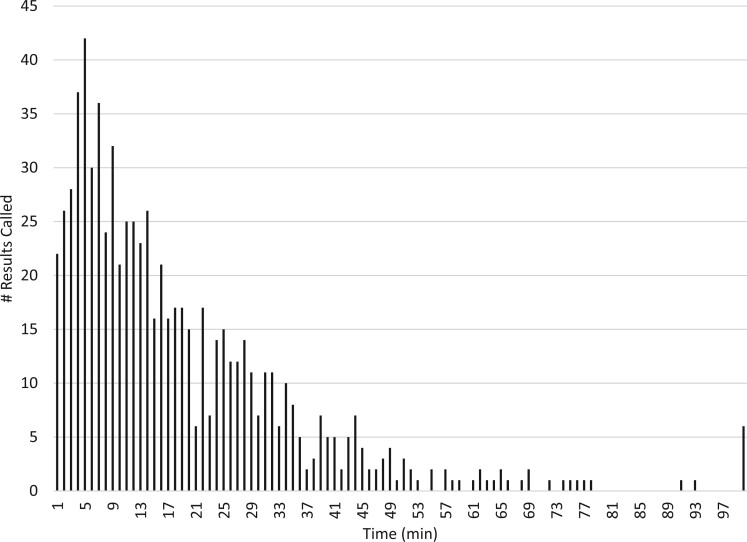
A snapshot of a given week of the number of critical results paged to the clinician as a function of time (min).

We first evaluated the process for alerting critical lab values. Figure 1 shows the workflow communication from lab services to the clinical team for processing critical lab results. We also looked at the number of pages for critical labs made by laboratory services to the teaching teams. Figure 2 is a snapshot of turnaround times (TAT) for a given week. As shown, most critical lab calls’ TAT are clustered within 10 minutes, but many are beyond this time frame and based on discussions with lab technicians, this was often attributed to calling the wrong clinician or not receiving a call back.

The aim of this project was to improve TAT by reducing the amount of time it takes for the provider to receive a critical lab result. The intention was to minimize changes to the existing workflow but incorporate a push notification via EPIC mobile application, Haiku. Another measure for this project was to reduce staffing burden in the clinical laboratory.

**Figure 3.**
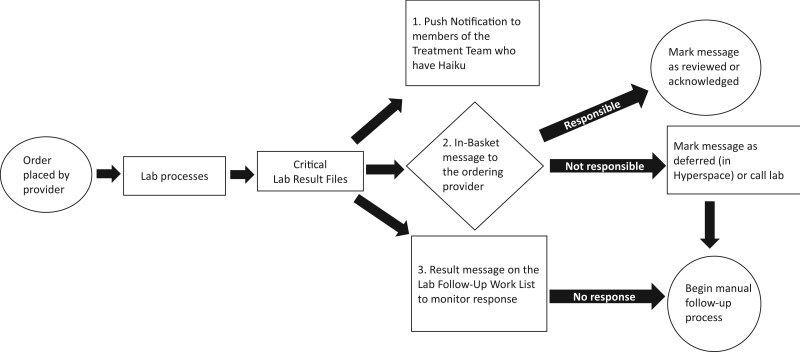
Workflow with implementation of push notifications. When the critical result files, a push notification is sent to the treatment team members in Haiku as well as in-basket message to the ordering clinician and on the lab services queue. If the responsible clinician receives the notification, they can review/acknowledge the result or defer. If no response within 10 min, the lab services defaults to manual process.

**Figure 4.**
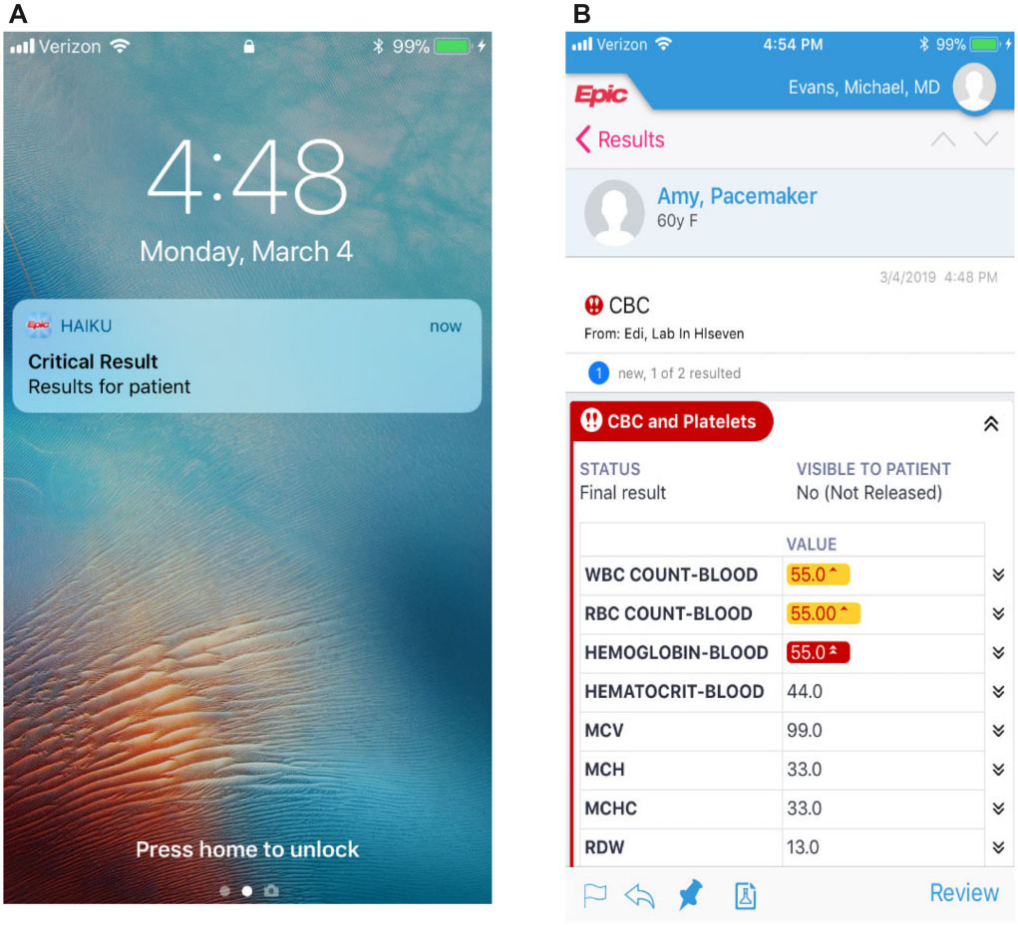
A, An example of how the push notification alert is sent to the clinician mobile device. B, When the critical lab is selected, a screenshot of an example lab that is viewed. The clinician then selects “review” to acknowledge the lab. This will also populate in the in-basket which can be reviewed or “deferred” in Epic. @2023 Epic Systems Corporation.

Figure 3 shows the workflow with the option for electronic acknowledgment of the critical lab result. The critical lab result’s status is reflected in the lab service technicians’ critical result queue. Acknowledged results reflect a status of “acknowledged.” Results not acknowledged within 10 minutes require lab services technicians to make a phone call to the provider. Figure 4 is an example of what the provider receives on their mobile device. Following receipt, the provider reviews and acknowledges the result, which is also accessible on the desktop version of EPIC. During the pilot period, we provided monthly reinforced education to the ICU treatment teams, including the use of tip sheets and direct feedback regarding their compliance with the electronic acknowledgment.

During the 3-month pilot, the electronic push notification process generated push notifications for 1414 critical lab results. Approximately 6% of these were electronically acknowledged (a total of 82). Table 1 shows a breakdown by month of those lab results acknowledged electronically following receipt of a push notification. As previously noted, each electronically acknowledged order represents 1 less page/call the lab service technician must make.

**Table 1.** The total number of critical labs and the number acknowledged by month with % acknowledged

	# critical labs	# results acknowledged	Acknowledged (%)
April	303	18	5.9
May	663	36	5.4
June	448	28	6.3

**Figure 5.**
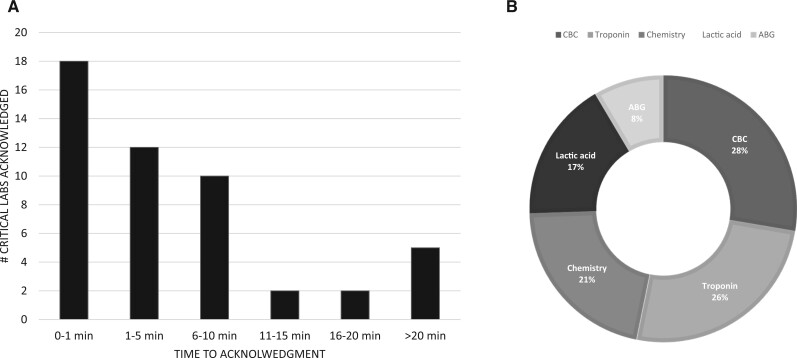
A, The # of critical labs acknowledged by the clinician as a function of time for the pilot project. B, A breakdown of the type of critical labs that were acknowledged by clinicians. ABG = arterial blood gas, CBC = complete blood count; chemistry includes basic metabolic and complete metabolic panels.

Figure 5A displays the quantity of critical labs electronically acknowledged by the TAT; you will notice that about 61% of results were acknowledged within 5 minutes and 81% within the first 10 minutes. Figure 5B shows the type of acknowledged critical labs.

## DISCUSSION

In the study by Verma et al,^10^ researchers used a cluster-randomization approach to study push notification of troponin values to smartphones. In the study, they found that physicians who received troponin push alerts discharged patients faster than those without notifications. In another study, push notification utilization improved patient flow for emergency department patients with suspected influenza.^11^ In this approach, the researchers used a notification platform to connect a notification to a frontline staff member via communication channels such as paging or e-mail. They found that a push notification improved throughput in the emergency department and provides a broader perspective of the utility of push notifications. Our study adds data on the advantages of push notification technology to the existing literature stream. Additionally, our study demonstrates that notifications can be easily integrated within the EHR.

During this pilot project, only a small percentage of results were electronically acknowledged. While this was lower than anticipated, the push notifications reduced the number of calls the lab services technicians had to make while allowing the ICU treatment team to have the critical lab results in real-time thus allowing them to better care for patients. There were several patients that had improved care because of the push notification and rapid acknowledgment.

Our study also demonstrated increased efficiency for the lab services technicians. Their reduction in phone calls allowed them to reallocate their resources to other tasks. Lastly, the transition, from a phone call to a push notification reduced the verbal disruptions for the ICU treatment team members receiving the escalation, thus improving the care teams’ efficiency too. Finally, the transition also reduced the risk of a verbal translation errors when the lab services technician relayed the critical lab result information.

### Limitations

One major limitation of this study was the turnover in providers from month to month which led to challenges around education. Our percentage of acknowledged notifications might also be low due to, in some instances, the care team provider was in the chart when the critical lab results were presented. The required action, to address the lab result, was in motion when the push notification was received; therefore, the provider dismissed the notification. Throughout this study, we incorporated a variety of education strategies including educating teams at each month’s orientation, direct feedback regarding the individual care team member’s performance and use of electronic acknowledgments, and quick tips sheets and periodic reminders. Despite the efforts, hand-offs to team members generated challenges; thus, the notifications were routed to the patient’s care team but not always the care team members on call when the result was received.

### Next steps

At the time of this pilot, nurses did not have a mobile device to get push notifications. Given their bedside presence, throughout the day, the notification should include them, thus allowing the whole care team to collaborate on the patient’s behalf. We plan to do this as a next step in this project. We also plan to expand the education to the Advanced Practice Providers as they are a constant workforce in our ICU settings and would minimize variation around education. In addition, we will consider speaking with direct users to elicit any recurrent barriers to develop strategies to increase the uptake of the push notification feature.

In summary, we show how this simple and familiar communication tool, that is, commonly used day-to-day, can also be used to improve efficiency in healthcare. Additional to the next steps outlined above, we also plan to expand to other ICU settings (surgical, cardiovascular unit) and ultimately all inpatient units and throughout the health system.

## CONCLUSION

We describe an innovative approach to push life-threatening critical lab notifications to clinicians’ handheld devices to reduce the time it takes to reach clinicians. Although we had a relatively small percentage of push notifications acknowledged, we believe that this led to improved patient care, reduced burden on our clinical lab services, and interruptions to clinicians.

## Data Availability

The data are available in Dryad Digital Repository, at DOI: 10.5061/dryad.5tb2rbp98.
